# Proporção entre o Volume Plaquetário Médio e a Largura de Distribuição das Plaquetas: O Parâmetro Plaquetário mais Eficaz no Fechamento do Canal Arterial

**DOI:** 10.36660/abc.20240635

**Published:** 2025-04-24

**Authors:** Ufuk Cakir, Cuneyt Tayman

**Affiliations:** 1 Division of Neonatology, Department of Pediatrics Health Sciences University Ankara Bilkent City Hospital Ankara Turquia Division of Neonatology, Department of Pediatrics, Health Sciences University, Ankara Bilkent City Hospital, Ankara – Turquia

**Keywords:** Volume Plaquetário Médio, Permeabilidade do Canal Arterial, Plaquetas, Recém-Nascido Prematuro

## Abstract

**Fundamento:**

O papel das plaquetas e dos índices plaquetários no fechamento do canal arterial em bebês prematuros ainda é controverso. Nosso objetivo foi determinar se a contagem de plaquetas e todos os índices relacionados às plaquetas são eficazes no fechamento do canal arterial patente (PDA).

**Objetivos:**

Características demográficas, morbidades de prematuridade e índices plaquetários foram comparados entre grupos com e sem PDA hemodinamicamente significativa (hsPDA).

**Métodos:**

Dados de prematuros com idade gestacional <30 semanas foram avaliados retrospectivamente. Todos os dados relacionados às plaquetas foram registrados a partir do hemograma completo nas primeiras 24 horas de todos os pacientes. A análise estatística foi realizada nos dados obtidos. Um valor de p <0,05 foi considerado estatisticamente significativo.

**Resultados:**

Um total de 1151 pacientes foram incluídos no estudo, incluindo 426 pacientes no grupo hsPDA e 725 pacientes no grupo não-hsPDA. Os parâmetros plaquetários volume plaquetário médio (VPM), VPM/plaquetcrit (PCT), VPM/largura de distribuição plaquetária (PDW), massa plaquetária (MP), largura de distribuição de hemácias (RDW) foram considerados significativamente menores no grupo hsPDA em comparação ao grupo não-hsPDA (p<0,001, p<0,001, p<0,001, p=0,015 e p<0,001, respectivamente). A razão VPM/PDW com o maior valor de AUC (0,748) foi considerada o parâmetro mais valioso na estimativa do fechamento da PDA.

**Conclusões:**

A relação VPM/PDW foi considerada o parâmetro mais valioso para a predição de hsPDA entre todos os índices plaquetários.

## Graphical abstract

**Figura Central:**
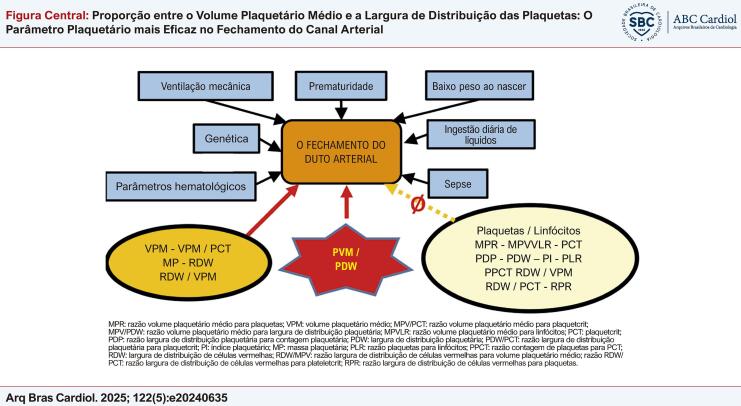
Proporção entre o Volume Plaquetário Médio e a Largura de Distribuição das Plaquetas: O Parâmetro Plaquetário mais Eficaz no Fechamento do Canal Arterial Resumo dos resultados do nosso estudo.

## Introdução

O canal arterial (DA) é um componente essencial do sistema circulatório durante a vida fetal. Ele conecta a artéria pulmonar principal à aorta descendente e desvia o débito ventricular dos pulmões para a aorta.^1^ A falha no fechamento do DA após o nascimento é definida como canal arterial patente (PDA) e geralmente está associada a um shunt da esquerda para a direita. Em bebês prematuros, o PDA hemodinamicamente significativo (hsPDA) pode levar a complicações cardiopulmonares graves, como sobrecarga de volume do ventrículo esquerdo, edema pulmonar, complacência pulmonar prejudicada e má perfusão abdominal-renal devido ao roubo ductal. Causa um aumento na morbidade prematura nos dias pós-natais subsequentes.^2^ O fechamento do DA é um enigma elusivo com fisiopatologia complexa e consequências clínicas que os neonatologistas enfrentam há décadas.^3^ Portanto, é crucial entender os mecanismos que contribuem para o fechamento do DA para fornecer cuidados especiais para prematuros em risco.^2^

Echtler et al. relataram pela primeira vez que as plaquetas foram eficazes no fechamento ductal em camundongos. Eles mostraram que as plaquetas foram direcionadas ao endotélio ductal em minutos após o nascimento, formando um tampão plaquetário e contribuindo para a remodelação ductal. Além disso, os autores relataram que a disfunção plaquetária afeta o fechamento do DA. Esses resultados mostram que as plaquetas desempenham um papel muito importante no fechamento do DA.^4^ Esses resultados lançam luz sobre estudos que avaliam a relação potencial entre a contagem de plaquetas e a presença de PDA no campo da neonatologia. No entanto, a significância clínica das plaquetas no fechamento do DA ainda é controversa e não foi confirmada in vivo.^2^ Estudos que avaliam a relação entre diferentes formulações de índices plaquetários, contagem plaquetária, funções plaquetárias e hsPDA são muito limitados. Os achados de estudos anteriores sobre índices plaquetários e PDA foram inconsistentes ou mesmo contraditórios, possivelmente devido às diferenças nos desenhos de estudo, protocolos de tratamento e definição de hsPDA.^5-10^ O aumento no número de estudos e a inclusão de outros parâmetros plaquetários forneceram mais dados para meta-análises. No entanto, a evidência para a relação entre plaquetas e PDA ainda é limitada devido à heterogeneidade clínica e estatística significativa entre diferentes estudos.^2,3^ Considerando a falta de estudos anteriores, não há estudo avaliando a relação entre todos os parâmetros relacionados às plaquetas e suas proporções entre si e PDA. Portanto, objetivamos avaliar a relação entre todos os parâmetros plaquetários e PDA, e a previsibilidade de possíveis parâmetros plaquetários relacionados a PDA.

## Métodos

### Desenho do estudo e seleção de pacientes

Nosso estudo foi conduzido retrospectivamente em bebês prematuros com idade gestacional (IG) <30 semanas que foram hospitalizados na unidade de terapia intensiva neonatal entre dezembro de 2018 e dezembro de 2022. Os dados foram obtidos dos prontuários médicos do hospital. Bebês com grandes anormalidades congênitas, cardiopatia congênita, asfixia perinatal, morte nos primeiros três dias após o nascimento (sem o diagnóstico de PDA) e idade gestacional ≥30 semanas foram excluídos do estudo. Características demográficas, morbidades devido à prematuridade e parâmetros de hemograma completo dos pacientes foram registrados. Os pacientes foram divididos em dois grupos: hsPDA e não hsPDA. A aprovação ética foi obtida do comitê de ética local do nosso hospital.

### Variáveis demográficas e resultados clínicos

Peso ao nascer (PN), IG, esteroides pré-natais, sexo, displasia broncopulmonar (DBP), hemorragia intraventricular (HIV, estágio ≥3), enterocolite necrosante (ECN, estágio >2), síndrome do desconforto respiratório (SDR), taxas de retinopatia da prematuridade (ROP), hsPDA e sepse foram registrados.

### Definição de morbidades relacionadas à prematuridade

DBP foi definida como pacientes que necessitavam de <30% de oxigênio (moderado), ≥30% de oxigênio ou suporte de pressão positiva (grave) em 36 semanas de idade corrigida pós-menstrual.^11^ Pacientes com HIV estágio ≥3 detectado por ultrassonografia transfontanelar foram definidos como HIV grave.^12^ Aqueles com ECN estágio ≥2, de acordo com achados clínicos e laboratoriais, foram registrados.^13^ Os pacientes foram definidos como RDS em caso de necessidade de surfactante.^14^ Pacientes com sepse clínica e cultura positiva foram inscritos.^15^ Pacientes diagnosticados com ROP no exame de retina e tratados foram registrados.^16^ Pacientes com e sem hsPDA foram inscritos.

### Determinação de persistência do canal arterial hemodinamicamente significativa

O exame ecocardiográfico Doppler foi rotineiramente realizado em recém-nascidos prematuros na septuagésima segunda hora após o nascimento com um transdutor GE Vivid 7 Pro, 10S (GE Healthcare, Salt Lake City, UT, EUA) por um cardiologista pediátrico. Com base nos achados ecocardiográficos, a hsPDA foi definida como um diâmetro ductal interno de ≥1,5 mm e/ou com uma relação átrio esquerdo (AE)/raiz aórtica (AO) ≥1,5. Os pacientes foram definidos como não-hsPDA se o diâmetro interno ductal fosse <1,5 mm e/ou a relação átrio esquerdo/raiz aórtica fosse <1,5, ou se nenhuma PDA fosse detectada. Os pacientes com hsPDA no acompanhamento ecocardiográfico foram tratados com ligadura farmacológica (anti-inflamatórios não esteroides) ou cirúrgica (se o tratamento médico falhasse).^17^

### Análise de Hemograma Completo e Índices de Plaquetas

Amostras de sangue foram obtidas por meio da veia periférica nas primeiras 24 horas após o parto.^7^ Amostras de sangue foram coletadas em tubos de ácido etilenodiaminotetracético (EDTA). A contagem de plaquetas (103 µ/L), o volume plaquetário médio (VPM, fL), a largura de distribuição plaquetária (PDW, %), o plaquetário crítico (PCT, %), a largura de distribuição de hemácias (RDW, %) e a contagem de linfócitos (103 µ/L) foram analisados pelo hemocitômetro automático Cell-Dyn 3700 (Abbott, Abbott Park, IL, EUA). Depois disso, foram contadas a razão volume plaquetário médio/plaquetas (MPR), razão volume plaquetário médio/plaquetário crítico (VPM/PCT), razão volume plaquetário médio/largura de distribuição plaquetária (VPM/PDW), razão volume plaquetário médio/linfócito (MPVLR), razão largura de distribuição plaquetária/contagem plaquetária (PDP), razão largura de distribuição plaquetária/plaquetário crítico (PDW/PCT), índice plaquetário (VPM x PDW/contagem plaquetária x PCT), massa plaquetária (MP: contagem plaquetária x VPM), razão plaquetária/linfócito (PLR), razão contagem plaquetária/PCT (PPCT), razão largura de distribuição de hemácias/volume plaquetário médio (RDW/VPM) e razão largura de distribuição de hemácias/plaquetas (RPR). Variáveis demográficas, desfechos clínicos e índices plaquetários foram comparados entre grupos com hsPDA e não-hsPDA.

### Análise Estatística

A análise estatística foi realizada com o programa Statistical Package for Social Sciences (SPSS), versão 20.0 (SPSS Inc, Chicago, IL, EUA). Métodos analíticos Kolmogorov-Smirnov e visual (histograma e gráficos de probabilidade) foram usados para avaliar a distribuição de todas as variáveis. A associação entre grupos para variáveis categóricas foi avaliada usando o teste qui-quadrado não pareado. O teste U de Mann-Whitney foi usado para a análise de variáveis contínuas, conforme apropriado. Variáveis contínuas com distribuição normal foram descritas como média e desvio padrão. Variáveis categóricas foram expressas como frequência. A análise das características operacionais do receptor (ROC) foi realizada para variáveis significativas. Após a análise ROC, a área sob a curva (AUC) e o intervalo de confiança (IC) de 95% da AUC foram calculados. Valores de limiar foram calculados para os parâmetros eficazes no fechamento ductal. Sensibilidade, especificidade, valor preditivo positivo (VPP) e valor preditivo negativo (VPN) foram determinados para valores de limiar. Valor de p < 0,05 foi considerado estatisticamente significativo. O tamanho da amostra foi introduzido para o desfecho primário como fechamento ductal; 117 pacientes em cada grupo teriam 80% de poder para detectar uma diferença de 25% entre os grupos (de 60 a 85%) na porcentagem de fechamentos permanentes, usando um teste χ2 bilateral corrigido pela continuidade em um nível de significância de 0,05,5

## Resultados

Durante o período do estudo, 1294 pacientes foram avaliados. De acordo com os critérios de exclusão do nosso estudo, 143 pacientes foram excluídos. Um total de 1151 pacientes foram incluídos no estudo com base nos critérios de inclusão. A IG média de todos os pacientes foi de 28,2 ± 1,2 semanas, e o PN médio foi de 1072 ± 231 g. 426 (37%) pacientes foram incluídos no grupo hsPDA, e 725 (63%) pacientes foram incluídos no grupo não-hsPDA. IG (28,1 ± 1,2 semanas) e PN (1067 ± 227 g) no grupo hsPDA foram semelhantes a IG (28,3 ± 1,1 semanas) e PN (1080 ± 232 g) no grupo não-hsPDA (p> 0,05). A frequência de DBP, HIV, RDS e ROP no grupo hsDPA foi significativamente maior do que no grupo não hsDPA (p<0,05). Os achados foram semelhantes entre os grupos em termos de esteroides pré-natais, gênero, ECN e sepse (p>0,05) (Tabela 1).

**Tabela 1:** – Variáveis demográficas e morbidades da prematuridade

Características do paciente	não-hsPDA (n=725)	hsPDA (n=426)	Valor-p
Semana gestacional, ^a^	28,3 ± 1,1	28,1 ± 1,2	0,101
Peso ao nascer, g^a^	1080 ± 232	1067 ± 227	0,074
Esteroide pré-natal, n (%)	530 (73,1)	298 (69,9)	0,303
Gênero masculino, n (%)	386 (53,2)	208 (48,8)	0,148
DBP, n (%)	62 (8,5)	115 (26,9)	<0,001*
HIV, n (%)	39 (5,4)	58 (13,6)	<0,001*
ECN, n (%)	15 (2.1)	10 (2,3)	0,763
RDS, n (%)	148 (20,4)	140 (32,8)	<0,001*
ROP, n (%)	43 (5,9)	55 (12,9)	<0,001*
Sepse, n (%)	297 (40,9)	170 (39,9)	0,741

^a^média ± desvio padrão. *P<0,05 foi considerado estatisticamente significativo. DBP: displasia broncopulmonar; HIV: hemorragia intraventricular; ECN: enterocolite necrosante; hsPDA: persistência do canal arterial hemodinamicamente significativa; SDR: síndrome do desconforto respiratório; ROP: retinopatia da prematuridade.

**Figura 1:**
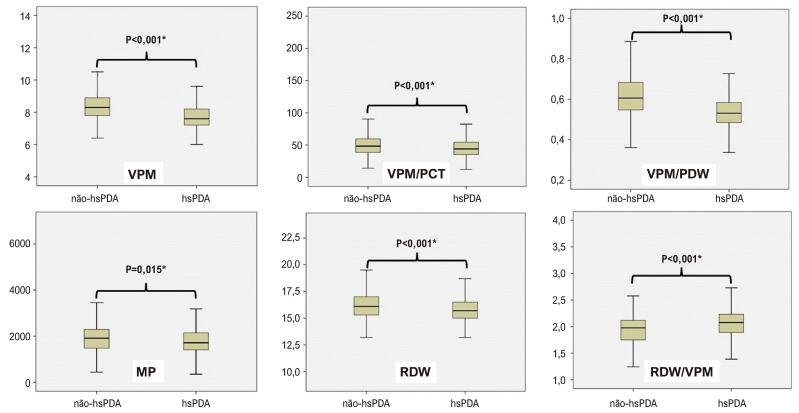
– Índices de persistência do canal arterial e plaquetas hemodinamicamente significativos. *P<0,05 foi considerado estatisticamente significativo. hsPDA: persistência do canal arterial hemodinamicamente significativa; VPM: volume plaquetário médio; VPM/PCT: razão volume plaquetário médio/plaquetário crítico; VPM/PDW: razão volume plaquetário médio/largura de distribuição plaquetária; MP: massa plaquetária; RDW: largura de distribuição de hemácias; RDW/VPM: razão largura de distribuição de hemácias/volume plaquetário médio.

Em termos de parâmetros plaquetários, incluindo VPM, VPM/PCT, VPM/PDW, MP e RDW, os valores foram significativamente menores no grupo hsPDA do que no grupo não-hsPDA (p<0,05). O valor RDW/VPM no grupo hsPDA foi significativamente maior do que no grupo não-hsPDA (p<0,05) (Tabela 2, Figura 1). Todos os outros parâmetros plaquetários (MPR, MPVLR, PCT, PDP, PDW, PDW/PCT, MP, PI, PLR, RDW/VPM, RPR, RDW/PCT e PPCT) foram semelhantes entre os grupos (p>0,05) (Tabela 2).

**Tabela 2:** – Parâmetros plaquetários de acordo com canal arterial patente hemodinamicamente significativo

Parâmetros	não-hsPDA (n=725)	hsPDA (n=426)	Valor-p
Contagem de plaquetas (10^3^ µ/L) ^a^	233 ± 85	243 ± 109	0,105
Linfócitos (10^3^ µ/L) ^a^	9,35 ±8,11	13,7 ± 10,37	0,204
MPR, ^a^	0,042 ± 0,02	0,040 ± 0,02	0,146
VPM, (fL) ^a^	8,40 ±0,93	7,65 ± 0,85	<0,001*
VPM/PCT, ^a^	52,40 ± 22,49	47,11 ± 20,65	<0,001*
VPM/PDW, ^a^	0,62 ± 0,10	0,53 ± 0,09	<0,001*
MPVLR, ^a^	1,40 ± 1,18	1,26 ± 1,19	0,057
PCT, (%) ^a^	0,186 ± 0,070	0,179 ± 0,056	0,094
PDP, ^a^	0,070 ±0,038	0,072 ± 0,053	0,569
PDW, (%)^a^	13,99 ±2,03	14,20 ± 2,10	0,103
PDW/PCT, ^a^	87,17 ± 38,55	87,67 ± 43,97	0,848
PI, ^a^	4,38 ± 3,86	4,00 ±3,55	0,273
MP^, a^	1944 ± 688	1834 ± 756	0,015*
PLR, ^a^	37,87 ± 29,95	40,49 ± 30,54	0,246
PPCT, ^a^	1306 ± 299	1312 ± 344	0,754
RDW, (%)^a^	16,23 ±1,56	15,71 ±1,71	<0,001*
RDW/VPM, ^a^	1,95 ± 0,25	2,07 ± 0,32	<0,001*
RDW/PCT, ^a^	101,01 ± 43,51	96,85 ± 45,50	0,129
RPR, ^a^	0,081 ± 0,042	0,079 ± 0,056	0,593

^a^média ± desvio padrão, *P<0,05 foi considerado estatisticamente significativo. hsPDA: persistência do canal arterial hemodinamicamente significativa; MPR: razão volume plaquetário médio/plaquetas; VPM: volume plaquetário médio; VPM/PCT: razão volume plaquetário médio/plaquetário crítico; VPM/PDW: razão volume plaquetário médio/largura de distribuição plaquetária; MPVLR: razão volume plaquetário médio/linfócito; PCT: plaquetário crítico; PDP: razão largura de distribuição plaquetária/contagem plaquetária; PDW: largura de distribuição plaquetária; PDW/PCT: razão largura de distribuição plaquetária/plaquetário crítico; PI: índice plaquetário; MP: massa plaquetária; PLR: razão plaquetária/linfócito; PPCT: razão contagem plaquetária/PCT; RDW: largura de distribuição de hemácias; RDW/VPM: razão largura de distribuição de hemácias/volume plaquetário médio; razão RDW/PCT: razão largura de distribuição de hemácias/plaquetário crítico; RPR: razão largura de distribuição de hemácias/plaquetário.

**Figura 2:**
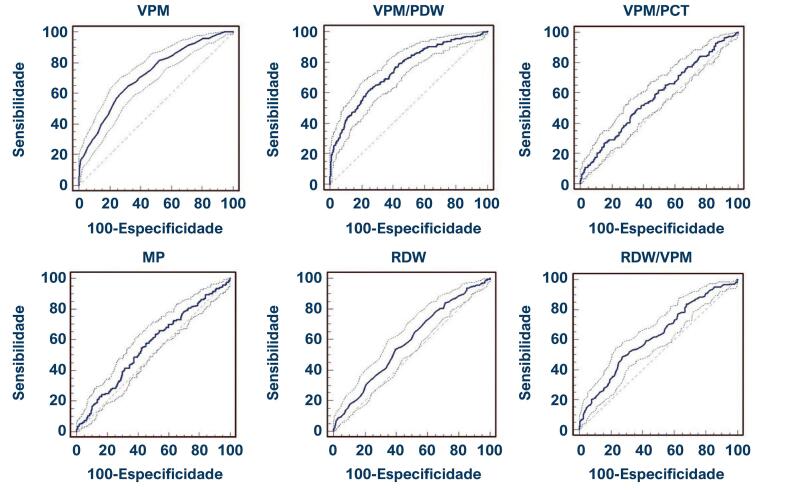
– Curvas características de operação do receptor para canal arterial patente hemodinamicamente significativo usando parâmetros plaquetários. VPM: volume plaquetário médio; VPM/PCT: razão volume plaquetário médio/plaquetário crítico; VPM/PDW: razão volume plaquetário médio/largura de distribuição plaquetária; MP: massa plaquetária; RDW: largura de distribuição de hemácias; RDW/VPM: razão largura de distribuição de hemácias/volume plaquetário médio.

A análise ROC foi realizada para avaliar a previsibilidade de VPM, VPM/PCT, VPM/PDW, MP, RDW e RDW/VPM. Valores de AUC, em ordem do maior para o menor: VPM/PDW, VPM, RDW/VPM, RDW, VPM/PCT e MP. Os resultados da análise ROC desses parâmetros (AUC, IC, sensibilidade, especificidade, VPP, VPN e valores-p) são mostrados na Tabela 3, e os gráficos são mostrados na Figura 2.

**Tabela 3:** – Análise da curva de operação do receptor dos parâmetros plaquetários

Parâmetros	AUC	Intervalo de confiança de 95%	Nível de corte	Sensibilidade (%)	Especificidade (%)	VPP (%)	VPN (%)	Valor-p
VPM (fL)	0,720	0,693-0,746	≤7,80	71	74	54	76	0,0001*
VPM/PCT	0,580	0,550-0,608	≤43,78	50	64	45	68	0,0001*
VPM/PDW	0,748	0,722-0,773	≤0,54	78	88	60	77	0,0001*
MP	0,561	0,532-0,592	≤1807	58	55	43	69	0,0004*
RDW (%)	0,587	0,557-0,615	≤15,70	54	60	44	69	0,0001*
RDW/VPM	0,621	0,592-0,649	>2,05	49	73	51	71	0,0001*

^*^P<0,05 foi considerado estatisticamente significativo. AUC: área sob a curva; VPP: valor preditivo positivo; VPN: valor preditivo negativo; VPM: volume plaquetário médio; VPM/PCT: razão entre volume plaquetário médio e plaquetcrit; VPM/PDW: razão entre volume plaquetário médio e largura de distribuição plaquetária; MP: índice de massa plaquetária; RDW: largura de distribuição de hemácias; RDW/VPM: razão entre largura de distribuição de hemácias e volume plaquetário médio.

## Discussão

Resultados semelhantes ou conflitantes foram obtidos em estudos anteriores avaliando a relação entre contagem de plaquetas, contagem de linfócitos, VPM, MPR, PCT, PDW, MP, PLR, RDW, RPR e hsPDA.^5-10,18-20^ A relação entre os valores de VPM/PDW, MPVLR, VPM/PCT, PDP, PDW/PCT, PI e RDW/VPM e hsPDA não foi avaliada antes. Em nossos resultados, nenhum efeito da contagem de plaquetas, contagem de linfócitos, MPR, PCT, PDW, PLR, MPVLR, PDP, PDW/PCT, PI e RPR no hsPDA foi detectado. Descobrimos que VPM, VPM/PDW, RDW, MP e VPM/PCT baixos e RDW/VPM altos foram associados ao hsPDA. A razão VPM/PDW com o maior valor de AUC (0,748) seguido por VPM (0,720) foram considerados os parâmetros mais valiosos na predição de hsPDA. Além disso, PPCT e RDW/PCT, que são parâmetros plaquetários, não foram avaliados anteriormente como fatores diagnósticos ou prognósticos em nenhuma doença. Pela primeira vez, descobrimos que PPCT e RDW/PCT não estavam associados a hsPDA.

O processo de fechamento do DA após o nascimento ocorre como resultado de uma série complexa de mecanismos. Como as plaquetas têm muitas interações inflamatórias e imunológicas com células endoteliais, a contagem de plaquetas e os parâmetros devem ser examinados para entender os mecanismos de fechamento ductal.^2^ Akar et al. e Meinarde et al. mostraram que a baixa contagem de plaquetas aumenta o risco de hsPDA.^10,21^ No entanto, paralelamente aos nossos resultados, outros estudos mostraram que o número de plaquetas não estava associado ao hsPDA.^8,9^ Embora a relação entre baixa contagem de plaquetas e hsPDA tenha sido demonstrada em camundongos, essa relação não foi finalizada em humanos devido a possíveis diferenças estruturais e fisiológicas ductais entre humanos e animais.^4^ Portanto, o efeito de outros índices plaquetários no DA deve ser investigado.

Acredita-se que a PCT, um fator relacionado às plaquetas que pode afetar o fechamento do DA, pode estar associada à hsPDA.^22^ No entanto, embora o valor da PCT possa ser menor na hsPDA, há evidências mostrando que esse valor não está relacionado à hsPDA.^5,8^ Bekmez et al. descobriram que a PCT era baixa e a RPR era alta na hsPDA, e relataram que a PDW não afetou o fechamento ductal.^5^ Em um estudo de recém-nascidos de 64 casos, foi relatado que a PLR foi significativamente maior no grupo hsPDA. Embora tenha sido relatado no mesmo estudo que a contagem de linfócitos era menor em pacientes com hsPDA, nossos resultados não corroboraram esses dados.^23^ No entanto, a diferença em nossos resultados pode ser devido ao fato de que a frequência de sepse foi semelhante em nossos grupos de estudo, e o número de pacientes e GA foram diferentes em outros estudos daqueles em nosso estudo.

Foi relatado que os valores de MPVLR e PLR são maiores em pacientes com aneurisma da aorta torácica no departamento de emergência em comparação ao grupo controle saudável.^24^ Com base nessa doença vascular, nenhum efeito desses parâmetros sobre hsPDA foi encontrado em nossos pacientes que foram avaliados para MPVLR e PLR. Foi relatado que os valores de PI, PDW/PCT, PDP e VPM/PCT de 40 pacientes pediátricos que morreram no hospital foram maiores do que os do grupo controle, e esses valores podem ser usados como um fator prognóstico.^25^ O efeito desses parâmetros sobre PDA não foi avaliado. Em nosso estudo, os valores de PI, PDW/PCT e PDP foram semelhantes entre os grupos, enquanto os valores de VPM/PCT foram significativamente menores no grupo hsPDA. Esses parâmetros podem ser usados para o acompanhamento e diagnóstico de hsPDA.

Resultados conflitantes foram relatados em relação ao efeito do MP no hsPDA.^6-10^ Por outro lado, o MP foi menor no grupo com hsPDA em nosso estudo. Um estudo anterior não relatou associação entre RDW e hsPDA. No entanto, descobrimos que o baixo RDW foi associado ao hsPDA. Os diferentes resultados entre RDW e hsPDA podem ser devidos ao fato de que o RDW é afetado pela inflamação.^20^ Em adultos, foi demonstrado que o RDW/VPM aumenta em correlação com a inflamação em pacientes com apendicite complicada.^26^ Em nosso estudo, o RDW/VPM foi significativamente maior em pacientes com hsPDA. Este resultado pode sugerir que este parâmetro pode mostrar inflamação no hsPDA e também pode ser usado no acompanhamento do hsPDA.

Em alguns estudos, nenhuma relação foi encontrada entre hsPDA e valor de VPM. Nesses estudos, o número de pacientes foi menor do que nossa população de pacientes, e eles tinham diferentes IG.^5-8,10^ Em um estudo com um número maior de pacientes, incluindo 481 bebês prematuros, uma correlação significativa foi encontrada entre baixo VPM e a presença de hsPDA. No mesmo estudo, o valor de AUC para VPM foi de 0,634, e o valor de corte foi <7,85 fL.^20^ No entanto, meta-análises relataram que VPM e PDW não estão associados com PDA. O principal problema dos estudos incluídos na meta-análise é que os pacientes têm heterogeneidade em termos de IG, PN, tempo de hemograma completo e número de pacientes.^1,3,22^ No entanto, embora tanto o VPM quanto o PDW forneçam informações sobre a atividade das plaquetas, a relação com a PDA ainda não está clara.^1^ Em nosso estudo, encontramos valores significativamente menores de VPM e VPM/PDW no grupo com hsPDA em comparação ao grupo sem hsPDA. O valor de VPM/PDW (nível de corte: ≤0,54) mostrou ser o parâmetro plaquetário com o maior valor de AUC e o maior poder preditivo de hsPDA. Além disso, o VPM (nível de corte: ≤7,8 fL) foi considerado o segundo valor com o poder preditivo de hsPDA. Nossos resultados lançarão luz sobre a relação entre VPM, PDW, atividade plaquetária e PDA.

Há uma deterioração das funções plaquetárias devido à imaturidade e doença crítica em bebês prematuros. Essas condições podem afetar o fechamento ductal.^6,7^ Portanto, usar parâmetros relacionados ao volume e distribuição plaquetária no hemograma completo pode ser uma abordagem facilmente acessível e de baixo custo para a previsibilidade de hsPDA. Em nosso estudo, foi descoberto que o menor volume de plaquetas, ou seja, o menor valor de VPM, aumentou o risco de hsPDA. A razão para isso é que plaquetas maiores (maior VPM) são mais propensas a reações protrombóticas e podem, em última análise, ser eficazes como um fator facilitador para o fechamento ductal.^6^ Além disso, plaquetas maiores e mais jovens são enzimaticamente e metabolicamente mais ativas do que as menores. Portanto, elas têm mais efeitos trombóticos.^7,9^ Em outras palavras, o VPM atua como um marcador de ativação plaquetária.^27^ Portanto, como em nossos resultados, à medida que o VPM diminui, o fechamento ductal pode ser afetado adversamente e o risco de hsPDA pode aumentar.

PDW é um indicador de liberação de plaquetas ativadas, variação no tamanho das plaquetas e ativação. Durante a ativação plaquetária, as plaquetas mudam de forma para obter uma superfície maior. Assim, o VPM e o PDW aumentam. Portanto, o uso combinado de VPM e PDW pode demonstrar de forma mais eficiente a ativação da coagulação.^22,27^ De acordo com a hipótese do papel das plaquetas no fechamento do DA, um maior grau de ativação plaquetária e, portanto, maior PDW é esperado em bebês sem PDA. No entanto, devido à heterogeneidade dos grupos mencionados em uma meta-análise publicada recentemente, o efeito do PDW no PDA não pôde ser demonstrado.^3^

Há resultados conflitantes em estudos que avaliam a relação entre VPM sozinho ou PDW sozinho e hsPDA.^6-10,20^ Em nosso estudo, o VPM foi encontrado menor em bebês com hsPDA. Embora o PDW tenha sido alto no grupo hsPDA, essa elevação não foi significativa. Portanto, de acordo com nossos resultados, o PDW sozinho não foi um marcador valioso para a previsibilidade de hsPDA. Antes do nosso estudo, o efeito do VPM/PDW no hsPDA era desconhecido. Descobrimos que o VPM/PDW foi superior e o parâmetro mais forte em comparação ao VPM sozinho na preditividade de hsPDA. Antić et al. relataram que o VPM/PDW foi semelhante entre os grupos em crianças com e sem apendicite complicada com inflamação grave.^26^ No entanto, Fan et al. mostraram que o VPM/PDW foi menor em pacientes com perfuração de úlcera duodenal em comparação ao grupo controle.^28^ Esses resultados mostraram que em algumas doenças, incluindo hsPDA, a forma e a distribuição das plaquetas, em vez de seu número, podem refletir mais efetivamente as funções plaquetárias.^1,22,27^

Além do número considerável de pacientes em nosso estudo, ele traz limitações devido à sua natureza retrospectiva e unicêntrica. No presente estudo, os parâmetros plaquetários foram calculados apenas nos valores do hemograma completo obtido nas primeiras 24 horas. Portanto, os índices plaquetários não puderam ser avaliados no acompanhamento e monitoramento da resposta ao tratamento da hsPDA. Além disso, nossos resultados são válidos para parâmetros nas primeiras 24 horas após o nascimento. Recomenda-se avaliar os parâmetros plaquetários em estudos prospectivos, randomizados e controlados com populações maiores.

## Conclusões

Determinamos pela primeira vez que o parâmetro plaquetário mais poderoso e novo para a preditividade de hsPDA foi VPM/PDW, que foi medido nas primeiras 24 horas de vida. VPM e PDW são dois parâmetros que podem ser medidos com dispositivos modernos. A razão VPM/PDW é um marcador importante, de baixo custo e rapidamente acessível que pode ser usado como um preditor de hsPDA. Portanto, nosso estudo pode ser uma pedra angular para estudos futuros sobre o valor prognóstico de VPM/PDW em bebês prematuros.
