# Supplementary material for: Proporção entre o Volume Plaquetário Médio e a Largura de Distribuição das Plaquetas: O Parâmetro Plaquetário mais Eficaz no Fechamento do Canal Arterial
Source: Arq Bras Cardiol. 2025 Apr 24;122(5):e20240635. [Article in Portuguese] doi: 10.36660/abc.20240635 (PMC12129483; doi:10.36660/abc.20240635)
Supplement: Translation

# Translation: Mean Platelet Volume to Platelet Distribution Width Ratio: The Most Effective Platelet Parameter in the Closer of Ductus Arteriosus

**DOI:** 10.36660/abc.20240635i

**Authors:** Ufuk Cakir, Cuneyt Tayman

**Affiliations:** 1 Türkiye Division of Neonatology, Department of Pediatrics, Health Sciences University, Ankara Bilkent City Hospital, Ankara – Türkiye

**Keywords:** Mean Platelet Volume, Patent Ductus Arteriosus, Blood Platelets, Premature Infant

## Abstract

**Background:**

The role of platelets and platelet indices in the closure of the ductus arteriosus in premature infants is still controversial. We aimed to determine whether the platelet count and all platelet-related indices are effective on the closer of patent ductus arteriosus (PDA).

**Objectives:**

Demographic characteristics, prematurity morbidities, and platelet indices were compared between groups with and without hemodynamically significant PDA (hsPDA).

**Methods:**

Data of premature infants with a gestational age of <30 weeks were evaluated retrospectively. All platelet-related data were recorded from the complete blood count in the first 24 hours of all patients. The statistical analysis was performed on the obtained data. A p-value of <0.05 was considered statistically significant.

**Results:**

A total of 1151 patients were included in the study, including 426 patients in the hsPDA group and 725 patients in the non-hsPDA group. Platelet parameters mean platelet volume (MPV), MPV/ plateletcrit (PCT), MPV/ platelet distribution width (PDW), platelet mass (PM), red cell distribution width (RDW) values were found to be significantly lower in the hsPDA group compared to the non-hsPDA group (p<0.001, p<0.001, p<0.001, p=0.015, and p<0.001, respectively). MPV/PDW ratio with the highest AUC value (0.748) was found to be the most valuable parameter in estimating the closer of PDA.

**Conclusions:**

The MPV/PDW ratio was found to be the most valuable parameter for the prediction of hsPDA among all platelet indices.

## Introduction

The ductus arteriosus (DA) is an essential component of the circulatory system during fetal life. It connects the main pulmonary artery to the descending aorta and diverts ventricular output from the lungs to the aorta.^1^ Failure of the closure of DA after birth is defined as patent ductus arteriosus (PDA) and is usually associated with a left-to-right shunt. In premature infants, hemodynamically significant PDA (hsPDA) can lead to serious cardiopulmonary complications such as left ventricular volume overload, pulmonary edema, impaired lung compliance, and abdominal-renal malperfusion due to ductal steal. It causes an increase in premature morbidity in the subsequent postnatal days.^2^ The closure of DA is an elusive enigma with complex pathophysiology and clinical consequences that neonatologists have faced for decades.^3^ Therefore, it is crucial to understand the mechanisms contributing to the closure of DA in order to provide special care for preterms at risk.^2^

Echtler et al. reported for the first time that platelets were effective in ductal closure in mice. They showed that platelets were directed to the ductal endothelium within minutes of birth, forming a platelet plug and contributing to ductal remodeling. Additionally, the authors reported that platelet dysfunction affects DA closure. These results show that platelets play a very important role in the closure of DA.^4^ These results shed light on studies evaluating the potential relationship between platelet count and the presence of PDA in the field of neonatology. However, the clinical significance of platelets in the closure of DA is still controversial and has not been confirmed in vivo.^2^ Studies evaluating the relationship between different formulations of platelet indices, platelet count, platelet functions, and hsPDA are very limited. Findings from previous studies on platelet indices and PDA have been inconsistent or even contradictory, possibly due to the differences in the study designs, treatment protocols, and definition of hsPDA.^5-10^ The increase in the number of studies and the inclusion of other platelet parameters provided more data for meta-analyses. However, evidence for the relationship between platelets and PDA is still limited due to significant clinical and statistical heterogeneity between different studies.^2,3^ Considering the lack of previous studies, there is no study evaluating the relationship between all platelet-related parameters and their ratios to each other and PDA. Therefore, we aimed to evaluate the relationship between all platelet parameters and PDA, and the predictivity of possible PDA-related platelet parameters.

## Methods

### Study Design and Patient Selection

Our study was conducted retrospectively in premature infants with a gestational age (GA) of <30 weeks who were hospitalized in the neonatal intensive care unit between December 2018 and December 2022. Data were obtained from the hospital’s medical records. Infants with major congenital abnormalities, congenital heart disease, perinatal asphyxia, death in the first three days after birth (without the diagnosis of PDA), and gestational age ≥30 weeks were excluded from the study. Demographic characteristics, morbidities due to prematurity, and complete blood count parameters of the patients were recorded. The patients were divided into two groups: hsPDA and non-hsPDA. Ethical approval was obtained from the local ethics committee of our hospital.

### Demographic variables and clinical outcomes

Birth weight (BW), GA, antenatal steroid, gender, bronchopulmonary dysplasia (BPD), intraventricular hemorrhage (IVH, stage ≥3), necrotizing enterocolitis (NEC, stage >2), respiratory distress syndrome (RDS), the rates of retinopathy of prematurity (ROP), hsPDA and sepsis were recorded.

### Definition of prematurity-related morbidities

BPD was defined as patients who were requiring <30% oxygen (moderate), ≥30% oxygen, or positive pressure support (severe) at 36 weeks of postmenstrual corrected age.^11^ Patients with stage ≥3 IVH detected by transfontanel ultrasonography were defined as severe IVH.^12^ Those with stage ≥2 NEC, according to both clinical and laboratory findings, were recorded.^13^ Patients were defined as RDS in case of need for surfactant.^14^ Patients with clinical and culture-positive sepsis were enrolled.^15^ Patients diagnosed with ROP in retinal examination and treated were recorded.^16^ Patients with and without hsPDA were enrolled.

### Determination of Hemodynamically Significant Patent Ductus Arteriosus

Doppler echocardiographic examination was routinely performed on preterm infants in the seventy-second hour after birth with a GE Vivid 7 Pro, 10S transducer (GE Healthcare, Salt Lake City, UT, USA) by a pediatric cardiologist. Based on echocardiographic findings, hsPDA was defined as an internal ductal diameter of ≥1.5 mm and/or with a left atrium (LA)/aortic root (AO) ratio ≥1.5. Patients were defined as non-hsPDA if the ductal inner diameter was <1.5 mm and/or the left atrium/aortic root ratio was <1.5, or if no PDA was detected. Patients with hsPDA in echocardiographic follow-up were treated with pharmacologic (non-steroidal anti-inflammatory drugs) or surgical ligation (if medical treatment failed).^17^

### Complete Blood Count Analysis and Platelet Indices

Blood samples were obtained via the peripheral vein within the first 24 hours of delivery.^7^ Blood samples were taken into ethylene diamine tetra acetic acid (EDTA) tubes. Platelet count (10^3^ µ/L), mean platelet volume (MPV, fL), platelet distribution width (PDW, %), plateletcrit (PCT, %), red cell distribution width (RDW, %) and lymphocytes count (10^3^ µ/L) were analyzed by the Cell-Dyn 3700 automatic hemocytometer (Abbott, Abbott Park, IL, USA). After that, mean platelet volume to platelet ratio (MPR), mean platelet volume to plateletcrit ratio (MPV/PCT), mean platelet volume to platelet distribution width ratio (MPV/PDW), mean platelet volume-to-lymphocyte ratio (MPVLR), platelet distribution width to platelet count ratio (PDP), platelet distribution width to plateletcrit ratio (PDW/PCT), platelet index (MPV x PDW/platelet count x PCT), platelet mass (PM: platelet count x MPV), platelet to lymphocyte ratio (PLR), platelet count to PCT ratio (PPCT), red cell distribution width to mean platelet volume ratio (RDW/MPV) and red cell distribution width to platelet ratio (RPR) were counted. Demographic variables, clinical outcomes and platelet indices were compared between groups with hsPDA and non-hsPDA.

### Statistical Analysis

Statistical analysis was performed with the Statistical Package for Social Sciences (SPSS), version 20.0 (SPSS Inc, Chicago, IL, USA) program. Analytical methods Kolmogorov-Smirnov and visual (histogram and probability plots) were used to evaluate the distribution of all variables. The association between groups for categorical variables was assessed using the unpaired Chi-Square test. Mann-Whitney U test was used for the analysis of continuous variables as appropriate. Continuous variables with normal distribution were described as mean and standard deviation Categorical variables were expressed as frequency. Receiver operating characteristics (ROC) analysis was performed for significant variables. After ROC analysis, the area under the curve (AUC) and the 95% confidence interval (CI) of the AUC were calculated. Threshold values were calculated for the parameters effective in ductal closure. Sensitivity, specificity, positive predictive value (PPV), and negative predictive value (NPV) were determined for threshold values. P value <0.05 was considered statistically significant. The size of the sample was introduced for the primary endpoint as ductal closure; 117 patients in each group would have 80% power for detecting a 25% between-group difference (from 60 to 85%) in the percentage of permanent closures, using a two-sided, continuity-corrected χ2 test at a significance level of 0.05.^5^

## Results

During the study period, 1294 patients were evaluated. According to the exclusion criteria of our study, 143 patients were excluded. A total of 1151 patients were included in the study based on the inclusion criteria. The mean GA of all patients was 28.2 ± 1.2 weeks, and the mean BW was 1072 ± 231 g. 426 (37%) patients were included in the hsPDA group, and 725 (63%) patients were included in the non-hsPDA group. GA (28.1±1.2 weeks) and BW (1067±227 g) in the hsPDA group were similar to GA (28.3±1.1 weeks) and BW (1080±232 g) in the non-hsPDA group (p>0.05). The frequency of BPD, IVH, RDS, and ROP in the hsDPA group was found to be significantly higher than in the non-hsDPA group (p<0.05). Findings were similar between the groups in terms of antenatal steroid, gender, NEC, and sepsis (p>0.05) (Table 1).

**Table 1:** – Demographic variables and prematurity morbidities

Patient’s characteristics	non-hsPDA (n=725)	hsPDA (n=426)	p value
Gestational week, ^a^	28.3 ± 1.1	28.1 ± 1.2	0.101
Birth weight, g ^a^	1080 ± 232	1067 ± 227	0.074
Antenatal steroid, n (%)	530 (73.1)	298 (69.9)	0.303
Male gender, n (%)	386 (53.2)	208 (48.8)	0.148
BPD, n (%)	62 (8.5)	115 (26.9)	<0.001*
IVH, n (%)	39 (5.4)	58 (13.6)	<0.001*
NEC, n (%)	15 (2.1)	10 (2.3)	0.763
RDS, n (%)	148 (20.4)	140 (32.8)	<0.001*
ROP, n (%)	43 (5.9)	55 (12.9)	<0.001*
Sepsis, n (%)	297 (40.9)	170 (39.9)	0.741

^a^mean ± standard deviation. *P<0.05 was considered statically significant. BPD: bronchopulmonary dysplasia; IVH: intraventricular hemorrhage; NEC: necrotising enterocolitis; hsPDA: hemodynamically significant patent ductus arteriosus; RDS: respiratory distress syndrome; ROP: retinopathy of prematurity.

In terms of platelet parameters, including MPV, MPV/PCT, MPV/PDW, PM, and RDW values were found to be significantly lower in the hsPDA group than in the non-hsPDA group (p<0.05). The RDW/MPV value in the hsPDA group was significantly higher than that in the non-hsPDA group (p<0.05) (Table 2, Figure 1). All other platelet parameters (MPR, MPVLR, PCT, PDP, PDW, PDW/PCT, PM, PI, PLR, RDW/MPV, RPR, RDW/PCT, and PPCT) were found to be similar between the groups (p>0.05) (Table 2).

**Table 2:** – Platelet parameters according to hemodynamically significant patent ductus arteriosus

Parameters	non-hsPDA (n=725)	hsPDA (n=426)	p value
Platelet count (103 µ/L) ^a^	233 ± 85	243 ± 109	0.105
Lymphocytes (103 µ/L) ^a^	9.35 ±8.11	13.7 ± 10.37	0.204
MPR, ^a^	0.042 ± 0.02	0.040 ± 0.02	0.146
MPV, (fL) ^a^	8.40 ±0.93	7.65 ± 0.85	<0.001*
MPV/PCT, ^a^	52.40 ± 22.49	47.11 ± 20.65	<0.001*
MPV/PDW, ^a^	0.62 ± 0.10	0.53 ± 0.09	<0.001*
MPVLR, ^a^	1.40 ± 1.18	1.26 ± 1.19	0.057
PCT, (%) ^a^	0.186 ± 0.070	0.179 ± 0.056	0.094
PDP, ^a^	0.070 ±0.038	0.072 ± 0.053	0.569
PDW, (%)^a^	13.99 ±2.03	14.20 ± 2.10	0.103
PDW/PCT, ^a^	87.17 ± 38.55	87.67 ± 43.97	0.848
PI, ^a^	4.38 ± 3.86	4.00 ±3.55	0.273
PM, ^a^	1944 ± 688	1834 ± 756	0.015*
PLR, ^a^	37.87 ± 29.95	40.49 ± 30.54	0.246
PPCT, ^a^	1306 ± 299	1312 ± 344	0.754
RDW, (%)^a^	16.23 ±1.56	15.71 ±1.71	<0.001*
RDW/MPV, ^a^	1.95 ± 0.25	2.07 ± 0.32	<0.001*
RDW/PCT, ^a^	101.01 ± 43.51	96.85 ± 45.50	0.129
RPR, ^a^	0.081 ± 0.042	0.079 ± 0.056	0.593

^a^mean ± standard deviation, *P<0.05 was considered statically significant. hsPDA: hemodynamically significant patent ductus arteriosus; MPR: mean platelet volume to platelet ratio; MPV: mean platelet volume; MPV/PCT: mean platelet volume to plateletcrit ratio; MPV/PDW: mean platelet volume to platelet distribution width ratio; PVLR: mean platelet volume-to-lymphocyte ratio; PCT: plateletcrit; PDP: platelet distribution width to platelet count ratio; PDW: platelet distribution width; PDW/PCT: platelet distribution width to plateletcrit ratio; PI: platelet index; PM: platelet mass; PLR: platelet to lymphocyte ratio; PPCT: platelet count to PCT ratio; RDW: red cell distribution width; RDW/MPV: red cell distribution width to mean platelet volume ratio; RDW/PCT ratio: red cell distribution width to plateletcrit ratio; RPR: red cell distribution width-to- platelet ratio;

**Figure 1:** – Hemodynamically significant patent ductus arteriosus and platelet indices. *P<0.05 was considered statically significant. hsPDA: hemodynamically significant patent ductus arteriosus; MPV: mean platelet volume; MPV/PCT: mean platelet volume to plateletcrit ratio; MPV/PDW: mean platelet volume to platelet distribution width ratio; PM: platelet mass index; RDW: red cell distribution width; RDW/MPV: red cell distribution width to mean platelet volume ratio.

ROC analysis was performed to evaluate the predictivity of MPV, MPV/PCT, MPV/PDW, PM, RDW, and RDW/MPV. AUC values, in order from highest to lowest: MPV/PDW, MPV, RDW/MPV, RDW, MPV/PCT, and PM. ROC analysis results of these parameters (AUC, CI, sensitivity, specificity, PPV, NPV, and p-values) are shown in Table 3, and graphics are shown in Figure 2.

**Table 3:** – Receiver operating curve analysis of platelet parameters

Parameters	AUC	95% Confidence interval	Cutoff level	Sensitivity (%)	Specificity (%)	PPV (%)	NPV (%)	p value
MPV (fL)	0.720	0.693-0.746	≤7.80	71	74	54	76	0.0001*
MPV/PCT	0.580	0.550-0.608	≤43.78	50	64	45	68	0.0001*
MPV/PDW	0.748	0.722-0.773	≤0.54	78	88	60	77	0.0001*
PM	0.561	0.532-0.592	≤1807	58	55	43	69	0.0004*
RDW (%)	0.587	0.557-0.615	≤15.70	54	60	44	69	0.0001*
RDW/MPV	0.621	0.592-0.649	>2.05	49	73	51	71	0.0001*

^*^P<0.05 was considered statically significant. AUC: area under curve; PPV: positive predictive value; NPV: negative predictive value; MPV: mean platelet volume; MPV/PCT: mean platelet volume to plateletcrit ratio; MPV/PDW: mean platelet volume to platelet distribution width ratio; PM: platelet mass index; RDW: red cell distribution width; RDW/MPV: red cell distribution width to mean platelet volume ratio.

**Figure 2:** – Receiver operating characteristic curves for hemodynamically significant patent ductus arteriosus using the platelet parameters. MPV: mean platelet volume; MPV/PCT: mean platelet volume to plateletcrit ratio; MPV/PDW: mean platelet volume to platelet distribution width ratio; PM: platelet mass index; RDW: red cell distribution width; RDW/MPV: red cell distribution width to mean platelet volume ratio.

## Discussion

Similar or conflicting results were obtained in previous studies evaluating the relationship between platelet count, lymphocyte count, MPV, MPR, PCT, PDW, PM, PLR, RDW, RPR, and hsPDA.^5-10,18-20^ The relationship between MPV/PDW, MPVLR, MPV/PCT, PDP, PDW/PCT, PI, and RDW/MPV values and hsPDA has not been evaluated before. In our results, no effect of platelet count, lymphocyte count, MPR, PCT, PDW, PLR, MPVLR, PDP, PDW/PCT, PI, and RPR on hsPDA was detected. We found that low MPV, MPV/PDW, RDW, PM, and MPV/PCT, and high RDW/MPV were associated with hsPDA. MPV/PDW ratio with the highest AUC value (0.748) followed by MPV (0.720) were found to be the most valuable parameters in predicting hsPDA. Additionally, PPCT and RDW/PCT, which are platelet parameters, have not been previously evaluated as diagnostic or prognostic factors in any disease. For the first time, we found that PPCT and RDW/PCT were not associated with hsPDA.

The closure process of DA after birth occurs as a result of a complex series of mechanisms. Since platelets have many inflammatory and immunological interactions with endothelial cells, platelet count and parameters should be examined to understand the mechanisms of ductal closure.^2^ Akar et al. and Meinarde et al. showed that low platelet count increases the risk of hsPDA.^10,21^ However, in parallel with our results, other studies have shown that the number of platelets was not associated with hsPDA.^8,9^ Although the relationship between low platelet count and hsPDA has been demonstrated in mice, this relationship has not been finalized in humans due to possible ductal structural and physiological differences between humans and animals.^4^ Therefore, the effect of other platelet indices on the DA should be investigated.

It is thought that PCT, a platelet-related factor that may affect DA closure, may be associated with hsPDA.^22^ However, while the PCT value may be lower in hsPDA, there has been evidence showing that this value is not related to hsPDA.^5,8^ Bekmez et al. found that the PCT was low and the RPR was high in hsPDA, and they reported that PDW did not affect the ductal closure.^5^ In a 64-case newborn study, it was reported that PLR was significantly higher in the hsPDA group. Although it was reported in the same study that lymphocyte count was lower in patients with hsPDA, our results did not support these data.^23^ However, the difference in our results may be due to the fact that the frequency of sepsis was similar in our study groups, and the number of patients and GA were different in other studies from those in our study.

It has been reported that MPVLR and PLR values are higher in thoracic aortic aneurysm patients in the emergency department compared to the healthy control group.^24^ Based on this vascular disease, no effect of these parameters on hsPDA was found in our patients who were evaluated for MPVLR and PLR. It has been reported that PI, PDW/PCT, PDP, and MPV/PCT values of 40 pediatric patients who died in the hospital were higher than the control group, and these values could be used as a prognostic factor.^25^ The effect of these parameters on PDA has not been evaluated. In our study, PI, PDW/PCT, and PDP values were similar between the groups, while MPV/PCT values were found to be significantly lower in the hsPDA group. These parameters may be used for the follow-up and diagnosis of hsPDA.

Conflicting results have been reported regarding the effect of PM on hsPDA.^6-10^ On the other hand, the PM was lower in the group with hsPDA in our study. A previous study reported no association between RDW and hsPDA. However, we found that low RDW was found to be associated with hsPDA. The different results between RDW and hsPDA may be due to the fact that RDW is affected by inflammation.^20^ In adults, it has been shown that RDW/MPV increases in correlation with inflammation in patients with complicated appendicitis.^26^ In our study, RDW/MPV was found to be significantly higher in patients with hsPDA. This result may suggest that this parameter may show inflammation in hsPDA and can also be used in the follow-up of hsPDA.

In some studies, no relationship was found between hsPDA and MPV value. In these studies, the number of patients was less than our patient population, and they had different GA.^5-8,10^In a study with a larger number of patients, including 481 premature infants, a significant correlation was found between low MPV and the presence of hsPDA. In the same study, the AUC value for MPV was 0.634, and the cutoff value was <7.85 fL.^20^ However, meta-analyses have reported that MPV and PDW are not associated with PDA. The main problem of the studies included in the meta-analysis is that the patients have heterogeneity in terms of GA, BW, time of complete blood count, and number of patients.^1,3,22^ However, although both MPV and PDW provide information about the activity of platelets, the relationship with PDA is still unclear.^1^ In our study, we found significantly lower MPV and MPV/PDW values in the group with hsPDA compared to the non-hsPDA group. MPV/PDW value (cutoff level: ≤0.54) was shown to be the platelet parameter with the highest AUC value and the highest predictive power of hsPDA. Moreover, MPV (cutoff level: ≤7.8 fL) was found to be the second value with the predictive power of hsPDA. Our results will shed light on the relationship between MPV, PDW, platelet activity, and PDA.

There is a deterioration of platelet functions due to immaturity and critical illness in premature infants. These conditions may affect ductal closure.^6,7^ Therefore, using parameters related to platelet volume and distribution in complete blood count may be an easily accessible and low-cost approach to hsPDA predictivity. In our study, it was found that the lower volume of the platelets, that is, the lower MPV value, increased the risk of hsPDA. The reason for this is that larger platelets (higher MPV) are more prone to prothrombotic reactions and may ultimately be effective as a facilitating factor for ductal closure.^6^ Furthermore, larger and younger platelets are enzymatically and metabolically more active than smaller ones. Therefore, they have more thrombotic effects.^7,9^ In other words, MPV acts as a platelet activation marker.^27^ Therefore, as in our results, as MPV decreases, ductal closure may be adversely affected and the risk of hsPDA may increase.

PDW is an indicator of activated platelet release, variation in platelet size, and activation. During platelet activation, platelets change shape to obtain a larger surface. Thus, MPV and PDW increase. Therefore, the combined use of MPV and PDW can more efficiently demonstrate coagulation activation.^22,27^ According to the hypothesis of the role of platelets in the closure of DA, a higher degree of platelet activation and, thus, higher PDW is expected in infants without PDA. However, due to the heterogeneity of the groups mentioned in a recently published meta-analysis, the effect of PDW on PDA could not be demonstrated.^3^

There are conflicting results in studies evaluating the relationship between MPV alone or PDW alone and hsPDA.^6-10,20^ In our study, MPV was found to be lower in infants with hsPDA. Although PDW was high in the hsPDA group, this elevation was not significant. Therefore, according to our results, PDW alone was not a valuable marker for the predictivity of hsPDA. Before our study, the effect of MPV/PDW on hsPDA was unknown. We found that MPV/PDW was superior and the strongest parameter compared to MPV alone in the predictivity of hsPDA. Antic et al. reported that MPV/PDW was similar between the groups in children with and without complicated appendicitis with severe inflammation.^26^ However, Fan et al. showed that MPV/PDW was lower in patients with duodenal ulcer perforation compared to the control group.^28^ These results showed that in some diseases, including hsPDA, the shape and distribution of platelets, rather than their number, may more effectively reflect platelet functions.^1,22,27^

In addition to the considerable number of patients in our study, it brings limitations due to its retrospective and single-centered nature. In the present study, the platelet parameters were calculated only on the values of the complete blood count taken within the first 24 hours. Therefore, platelet indices could not be evaluated in the follow-up and monitoring of the response to the treatment of hsPDA. Moreover, our results are valid for parameters in the first 24 hours after birth. It is recommended to evaluate platelet parameters in prospective, randomized controlled studies with larger populations.

## Conclusions

We determined for the first time that the most powerful and new platelet parameter for the predictivity of hsPDA was MPV/PDW, which was measured in the first 24 hours of life. MPV and PDW are two parameters that can be measured with modern devices. MPV/PDW ratio is an important, low-cost, and rapidly accessible marker that can be used as a predictor of hsPDA. Therefore, our study may be a cornerstone for future studies on the prognostic value of MPV/PDW in premature infants.
